# The Relations of Physicians and Druggists

**Published:** 1875-07

**Authors:** Ferdinand King


### THE RELATIONS OF PHYSICIANS AND DRUGGISTS.

*Read before the Middle Georgia Medical Society, at Forsyth, Georgia, May 19th*, 1875.

By FERDINAND KING, M.D.

Prompted by a desire to contribute everything in my power to the advancement of physic and pharmacy, I come before you to-day with a few suggestions on the relations of physicians and druggists. I say suggestions, because I am cognizant of the fact that among this intelligent body of medical men are some who have been converts of Æsculapius for more than a quarter of a century. But with a due regard for the experince of these old practitioners of medicine, I think I may safely say, that during a service of more than seven years behind the druggist's counter, I have learned some facts that are worthy of the serious consideration of both professions.

In the application of substances for the relief of disease, or in the practice of medicine, the two professions are brought prominently into action. These two professions—physic and pharmacy—originally constituted the practice of medicine. The fathers of medicine, Galen and Hippocrates, practised, as do many of their successors, both professions, and may with equal propriety be considered the fathers of physic and pharmacy. With the wonderful discoveries of chemistry, the increase of medicinal substances and compounds, by the aid of the microscope, a closer study of the structure of the human body, and of the causes of disease, the scope of pharmacy and physic has been so extended as to embrace in medicine much more than one person can master and practice to advantage. As a natural result of these facts, there is and has been a tendency toward the separation of the two professions, which has been already carried out, to a greater or less extent, in some localities. Should it be desirable that a permanent separation be made, the proper basis would doubtless be to secure to each its peculiar functions and privileges without conflicting in any way with the other. The peculiar function and privilege of the physician is to supply medical advice. The peculiar function and privilege of the druggist or pharmaceutist is to supply medicine. The question of education and qualification, while of the greatest importance to both professions, should only be considered and acted on by each for itself. Neither has the right to make any qualifications for, or set in judgment on, the peculiar practices of the other. To the physician anyone who supplies medicine is a pharmaceutist; and to the pharmaceutist anyone who writes a prescription is a physician. With these fundamental principles thoroughly understood and practised, a separation, in a business point of view, can be easily effected that would result in permanent good and equal prosperity to both. And it is only by adhering to general principles that harmony and co-operation can be secured. Being twin sisters, as it were, the two professions of medicine should work together hand in hand, for their own and the public good. Being of the same parentage, and sharing equally in the same venerable traditions and history, neither has anything to gain by claiming to be the superior of the other. The physician who prescribes any particular standard preparation or unofficinal elixir is as guilty as the pharmaceutist who recommends a preparation of any particular manufacturer. Many of our most prominent physicians do not hesitate to prescribe the numerous fancy elixirs, syrups, sugar-coated pills, etc., that are being put upon the market by enterprising pharmaceutists of New York and other large cities, while they spurn with contempt the idea of prescribing proprietary, or, as more commonly called, patent medicines. Why should they do this? In so doing they are guilty of what might be considered in our code a breach of medical ethics, because these elixirs are prepared by processes that are patented, making them really proprietary articles. The physician should do everything in his power to aid his step-brother, the pharmaceutist, in crushing out both the patent medicine or real quackery, as well as the elixir or *quasi* quackery. The physicians and pharmaceutists can do this if they will only work together. I make bold to assert that there is only one way of successfully eradicating these evils; that is by fighting quackery with its own weapons. Let every pharmaceutist prepare and always keep on hand a well-appointed stock of so-called patent medicines of his own make, using recipes furnished him by efficient and competent physicians; and let him recommend these to his customers, as occasion permits. Instead of pushing villainous concoctions, whose composition he has a right to infer is fraudulent and falsely stated by the manufacturers, the pharmaceutist should offer his preparations as a substitute, which he can conscientiously indorse as a suitable medicine for that particular object which the consumer is intent on having a remedy for. It is within the scope of every intelligent pharmaceutist to furnish better and cheaper compounds, of a simpler nature, than the nostrum-makers do; and, being inspired by humane and honorable motives, the pharmaceutist has it in his power, under such circumstances, to satisfy the patient by selling him his money's worth, and thereby leave the voracious quack out in the cold, where he duly belongs, and where he will eventually find his proper level, and betake himself to more congenial and remunerative employment. These preparations should be put up in similar and equally elegant style to most of the more popular patent compounds now extant, with full directions on the label or wrapper; and the medicines should not only be elegantly put up, but pleasant to the taste. Among the list should be blood purifiers, (made from stillingia, yellow-dock, iodide potassium, etc.,) liniments, plasters, pills, tonics, ague remedies, etc. Any competent pharmaceutist can coat his pills, making them as elegant as the specialist. As an offset to the quackman's almanac, the pharmaceutist can wrap about his bottles a catalogue of preparations containing a calendar and other useful information in regard to the various simple elements generally used in domestic practice. Physicians should by no means take offense at such a departure in pharmacy, but really sanction and indorse the course as an actual advance in the eradication of the most pernicious quackery.

The second style and less persistent of offensive quacks is the elixir and elegant pharmacy specialists. Their field of operation is among the doctors, to whom their nice young men pay marked attention, and to whom the employers of these agents send seductive missives on the wonderful progress and marvelous achievements in combining all sorts of incompatabilities and impossibilities. The remedy for this evil is within the reach of every physician. These elixirs, as we remarked before, are made by patent processes, so don't prescribe them. But I have already taken up more space with the "patent" subject than I intended, so I will hasten on.

We next come to speak of physicians and druggists in their daily transactions or business intercourse. It is of the utmost importance that physicians, in writing prescriptions, exercise the greatest possible caution; for many fatal mistakes are traceable to this lack of prudence. They should make every abbreviation complete, and thereby save the pharmaceutist the trouble of sending to them so often to learn whether, when they write "hyd. chl.," they want calomel or chloral hydrate. But we consider it of more importance, if possible, that they pay special attention in making the ℥ and ʒ marks. In fact, considering the seriousness of this matter of characters, the Academy of Medicine at Richmond, Va., at a late meeting in that city, recommended that physicians dispense with the time-honored ʒ mark, and substitute in its stead the Greek capital (Δ) delta, with the shape of which you are all familiar. They also suggested that the letters P. C. (standing for *prœter consuetudinem*) be written by the physician on the left of and on the same line with the name of any potent ingredient, when purposely prescribed in doses larger than ordinarily used. When from any indiscretion on the part of the physician a mistake is made by the pharmaceutist, even if a fatal one, the former should come promptly to the assistance of the latter, receiving his due proportion of public censure, instead of remaining securely shielded by high standing, or the euphonious title of M.D. I remember myself, while a clerk in a New York store, having a prescription, written by a leading physician of the city, handed me at midnight to be dispensed. On examination, I discovered what I considered a mistake, in that he had written *seven ounces*, when he intended *seven drachms*. But, as I had no boy in the store, at such an unseasonable hour, whom I could send for a correction, and knowing the writer to be a dogmatic personage, who had very little sympathy for clerks, I prepared the mixture, which filled a *twelve ounce bottle*. Though he had written *seven ounces*, there was no incompatibility, nor was the dose too much as prescribed. I simply wanted to be a little contrary, and pay him in his own coin for some of his former treatment. On the back side of the prescription I wrote "too much catechu, but not enough caution." The next day, while my employer was attending the dispensing department of the store, and I was in the work-room, or laboratory, preparing some fancy "elixirs" for the doctors away down South, the old M.D. of the night before rushed into the store, telling my "boss" that I had made a huge mistake, which, though not fatal, showed that I was not sufficiently careful to occupy the responsible position I then filled. Of course I felt "spotted," to use a horse phrase, yet I felt tolerably safe. With the sacred, 86,492—I can't forget it—in his hand the boss, followed by the exasperated M.D., repaired to the file, where in a few moments they learned all. I was right, and the old M.D. wrong and willing to sell out for a sixpence. I cite the above as only one among many mistakes that have come under my personal observation. Such should certainly be charged to the physician, and placed to the credit of the pharmaceutist.

While showing up the M.D.'s I will also tell a thing or two on the pill-rollers, as I know all their little tricks, having learned them from experience. Among the many bad things they are guilty of, none should be more seriously condemned than the habit some druggists have of telling their customers what articles the prescription contains, when it is written in technicalities. Furthermore, they sometimes tell the effects that will be produced by the administration of the medicine. Another and even more pernicious habit is that of prescribing across the counter. Some pharmaceutists do this with a degree of solemnity that would shame the ghost of Hippocrates. How competent they are to do this, when they don't know whether the pill they see fit to administer finds its way into the stomach through the æsophagus or trachea! They even think the receptacle of food, etc. is in the hypogastric region.

But for fear the physicians fail to get their due proportion, I will refer to another hurtful habit on their part. They are often guilty of telling their patients how much their prescriptions will cost. This they should not do, for various reasons; the most practicable of which is that it exhibits a seeming disposition on their part to dictate to the pharmaceutist—in other words, to price his goods for him.

Finally, gentlemen, I again repeat it : We can never eradicate these growing evils until we shall have formed ourselves into a solid, lasting and mutual compact, each working for the other's good. The druggists of Georgia should meet in convention, and organize a Pharmaceutical Association, which should work in co-operation with the Georgia Medical Association. By so doing the many evils and inaccuracies enumerated in this short paper would be corrected.

